# The Role of Sodium-Glucose Cotransporter-2 Inhibitors in Heart Failure Management: The Continuing Challenge of Clinical Outcome Endpoints in Heart Failure Trials

**DOI:** 10.3390/pharmaceutics15041092

**Published:** 2023-03-29

**Authors:** Luxi Ji, Mudit Mishra, Bart De Geest

**Affiliations:** Centre for Molecular and Vascular Biology, Catholic University of Leuven, 3000 Leuven, Belgium; luxi.ji@student.kuleuven.be (L.J.); drmuditm@gmail.com (M.M.)

**Keywords:** heart failure, heart failure with preserved ejection fraction, heart failure with reduced ejection fraction, sodium-glucose cotransporter-2 (SGLT2) inhibitors, clinical outcome endpoints, composite endpoints

## Abstract

The introduction of sodium-glucose cotransporter-2 (SGLT2) inhibitors in the management of heart failure with preserved ejection fraction (HFpEF) may be regarded as the first effective treatment in these patients. However, this proposition must be evaluated from the perspective of the complexity of clinical outcome endpoints in heart failure. The major goals of heart failure treatment have been categorized as: (1) reduction in (cardiovascular) mortality, (2) prevention of recurrent hospitalizations due to worsening heart failure, and (3) improvement in clinical status, functional capacity, and quality of life. The use of the composite primary endpoint of cardiovascular death and hospitalization for heart failure in SGLT2 inhibitor HFpEF trials flowed from the assumption that hospitalization for heart failure is a proxy for subsequent cardiovascular death. The use of this composite endpoint was not justified since the effect of the intervention on both components was clearly distinct. Moreover, the lack of convincing and clinically meaningful effects of SGLT2 inhibitors on metrics of heart failure-related health status indicates that the effect of this class of drugs in HFpEF patients is essentially restricted to an effect on hospitalization for heart failure. In conclusion, SGLT2 inhibitors do not represent a substantial breakthrough in the management of HFpEF.

## 1. Introduction

Heart failure is the cardiovascular epidemic of the 21st century [1]. This syndrome is a growing public health problem, the leading cause of hospitalization, and a major cause of mortality [2]. It is the pathophysiological state in which cardiac dysfunction is responsible for failure of the heart to pump blood at a rate commensurate with the requirements of metabolizing tissues, or when sufficient cardiac output can only be generated at the expense of elevated filling pressures. The ejection fraction is normally distributed in the general population but has a bimodal distribution among patients with incident heart failure [3]. A distinction is made between heart failure with reduced ejection fraction (HFrEF) (ejection fraction ≤ 40%), heart failure with mid-range or mildly reduced ejection fraction (40.1–49.9%) (HFmrEF), and heart failure with preserved ejection fraction (HFpEF) (ejection fraction ≥ 50%) [4]. Heart failure is the consequence of structural or functional abnormalities of the heart. However, the diagnosis of heart failure requires the presence of clinical symptoms. Cardiac dysfunction and structural abnormalities of the heart are insufficient for the diagnosis of heart failure and can only corroborate a diagnosis, which remains today, even in the presence of elevated natriuretic peptides, a clinical diagnosis.

Prevalence of heart failure in the general population is 1–2% [5]. The prevalence of heart failure increases with age. The prevalence of heart failure is 7% in the age group 75–84 years and over 10% in those older than 85 years [6,7]. The incidence of heart failure approaches 5–10 per 1000 persons per year [8]. The age-adjusted incidence of heart failure appears to stabilize or is even declining [9,10]. Nevertheless, as the population ages, heart failure will continue to be a growing problem [9,10]. Heart failure is the dominant cause of hospital admissions and accounts for 1% to 2% of all hospitalizations [10,11,12,13]. Heart failure has a poor prognosis [14,15]. Based on the analysis of one large clinical register containing data on 39,982 patients who were admitted for heart failure, 18,299 (46%) patients had HFpEF, 3285 (8.2%) patients had HFmrEF, and 18,398 (46%) had HFrEF [16]. Overall, the median survival was 2.1 years. All three groups had similar 5-year mortality (HFpEF 75.7%; HFrEF 75.3%; HFmrEF 75.7%) [16]. Although these data may be susceptible to selection bias, these figures are a clear indication of the severity of the heart failure syndrome.

The data in the previous paragraph are not in contradiction with the fact that the natural history of HFrEF is more severe than the natural history of HFpEF. The natural history is the progression of a disease process over time, in the absence of treatment. It ends with death or resolution of the disease but can also persist in the presence of reduced function or disability of the patient. Inhibition of the renin-angiotensin-aldosterone system, ß-receptor blockade, and neprilysin inhibition by sacubitril improve survival and decrease hospitalizations in patients with HFrEF [17,18,19]. In contrast to these advances in the treatment of HFrEF, drug strategies with strong evidence in HFrEF have been proven to be unsuccessful in HFpEF. Therefore, the need for disease-modifying therapies with novel mechanisms of action continues to be required for further progress, in particular for the effective treatment of HFpEF. The introduction of sodium-glucose cotransporter-2 (SGLT2) inhibitors in heart failure management has been regarded by many as a major advance in the treatment of heart failure, particularly in the treatment of HFpEF [20]. As will be discussed in this review, this view is at least unbalanced.

SGLT2 inhibitors were initially developed as novel oral glucose-lowering agents that improve glycemic control independently of insulin secretion by inducing glucosuria. The SGLT2 inhibitors also result in a modest reduction in blood pressure and body weight. The pharmacology of this class of anti-diabetic agents is discussed in detail in Section 2. Given the relationship between type 2 diabetes and cardiovascular disease and the fact that certain antidiabetic drugs increase cardiovascular risk, the evaluation of cardiovascular safety was required during drug development based on regulations in the United States and Europe [21]. Section 3 lists important large-scale cardiovascular safety trials of currently approved SGLT2 inhibitors in type 2 diabetic patients. The surprising result of these trials was that these drugs not only do not cause harm, but may have cardioprotective effects and may counteract the development of heart failure. The primary outcome measure in definitive phase III clinical trials should be a clinical event relevant to the patient or an endpoint that measures directly how a patient feels, functions, or survives, where function refers to the patients’ ability to perform activities in their daily lives [22]. The application of this definition in the field of heart failure is complex. Therefore, the therapeutic goals and meaningful endpoints of heart failure trials are reviewed in Section 4, in order to provide a framework for the interpretation of the available evidence on SGLT2 inhibitors in heart failure, which are illustrated in Section 5. In Section 6, we discuss the interchangeability of SGLT2 inhibitors in the treatment of chronic heart failure. Hereto, we analyze the clinical trials from the perspective of methodological heterogeneity and clinical heterogeneity and evaluate potential within-class variations in the effects of these drugs. Perspectives, future directions, and conclusions are presented in Section 7. This sequence of sections serves the ultimate aim of this review, which is to provide a balanced view on the value of SGLT2 inhibitors in chronic heart failure management by carefully analyzing the results of major clinical HFrEF and HFmrEF/HFpEF trials from the perspective of the validity of the primary composite endpoint applied in these investigations.

## 2. Pharmacology of SGLT2 Inhibitors

### 2.1. Pharmacodynamics and Pharmacokinetics

In the kidney and the intestine, glucose transport across epithelial cells occurs via active transport, which is achieved by sodium-glucose cotransporter proteins [23]. The absorption of glucose molecules happens against the concentration gradient across the apical membrane, powered by the sodium co-transport down its concentration gradient, which is maintained by Na^+^/K^+^ ATPase [23]. The increased intracellular concentration of glucose enables the subsequent passive transfer of glucose across the basolateral membrane.

In the kidneys, 100% of the filtered glucose in the glomerulus is reabsorbed under physiological conditions along the nephron. SGLT has two main types. SGLT1 accounts for glucose absorption from the small intestine and to a minor extent also contributes to glucose reabsorption in the kidney, while SGLT2 is responsible for the reabsorption of most of the filtered glucose in the kidney. SGLT1 is an integral membrane protein that has low-capacity and high-affinity characteristics and is mainly expressed in the intestine [24]. In type 2 diabetic patients, SGLT1 expression in the intestine is 3- to 4-fold higher compared to non-diabetic patients, suggesting an increased capacity for glucose absorption [25]. SGLT2 macromolecules are sodium-dependent glucose transport proteins with high-capacity and low affinity. SGLT2 transporters are primarily expressed in the S1 segment of the proximal renal tubule. Approximately 90% of the glucose reabsorption in the kidney is accomplished by this SGLT2 transporter group [26]. SGLT1 is expressed in the S3 segment of the proximal tubule and accounts for 10% of renal glucose reabsorption [27].

In healthy individuals, the renal tubules are capable of reabsorbing nearly 100% of filtered plasma glucose. Hyperglycemia results in an increased amount of filtered glucose, which is reabsorbed until the maximal capacity for the reabsorption of tubules is reached. The renal threshold of glucose refers to a particular plasma glucose concentration, beyond which glucose is eliminated in the urine (i.e., glucosuria) [28]. In diabetic patients, the renal glucose reabsorption capacity is elevated, which probably reflects an adaptation to chronic exposure to a high load of filtered glucose [29]. Preclinical studies have indeed revealed the possibility of SGLT adaptation to chronic hyperglycemia in rats [30]. The induction of diabetes in these rats stimulated mRNA expression of SGLT2 via hepatocyte nuclear factor-1α, which directly controls SGLT2 gene expression [30].

Phlorizin, known since the nineteenth century for causing glucosuria, was later proven to be a potent inhibitor of SGLT1 and SGLT2 [31,32]. This phlorizin-induced glucosuria was associated with the normalization of glycemia in alloxan-induced diabetic rats without hypoglycemia and weight gain, which are known side-effects of insulin therapy [30]. Discontinuation of phlorizin application resulted in the restoration of hyperglycemia and insulin resistance [33]. However, the non-selectivity of phlorizin leads to unavoidable side-effects such as diarrhea due to the blockage of intestinal glucose uptake [34]. Consequently, the development of highly selective SGLT2 inhibitors as an anti-diabetic treatment has been pursued [35].

Currently, four SGLT2 inhibitors are licensed by both the European Medicines Agency (EMA) and the United States Food and Drug Administration (FDA): canagliflozin, dapagliflozin, empagliflozin, and ertugliflozin. They differ pharmacologically from each other mainly by the relative selectivity for SGLT2 over SGLT1, which amounts to approximately 260-fold for canagliflozin [36], 1200-fold for dapagliflozin, 2700-fold for empagliflozin [37], and 2200-fold for ertugliflozin [38], respectively. Pharmacokinetically, canagliflozin, dapagliflozin, empagliflozin, and ertugliflozin show very similar characteristics. For example, they have a rather long elimination half-life within the range of 11–18 h, allowing once-daily administration. All four drugs are rapidly absorbed following oral administration, do not lead to the formation of active metabolites, and have a limited renal excretion [39,40,41,42,43].

### 2.2. Mechanism of Glycemic Control and Effect on Body Weight

Multiple clinical trials have demonstrated the capacity of SGLT2 inhibitors to improve glycemic control in type 2 diabetes. When SGLT2 inhibitors were used as monotherapy or in addition to other anti-hyperglycemic therapies in type 2 diabetic patients, their fasting plasma glucose and glycated hemoglobin (HbA1c) were significantly reduced [44,45]. SGLT2 inhibition blocks a great part of glucose reabsorption within the kidney and in this way lowers the renal threshold for glucose. The increased urinary glucose excretion effectively reduces plasma glucose [37,45,46,47]. Furthermore, SGLT2 inhibitors also reduce hyperglycemia-induced glucotoxicity, which refers to the ability of excess glucose to impair both insulin secretion and insulin action, leading to a vicious cycle of worsening glycemic control in diabetes mellitus. In other words, SGLT2 inhibitors improve insulin sensitivity and enhance β-cell function [48,49].

The SGLT2 inhibitor-induced elevated urinary glucose excretion led to a loss of approximately 75 g of glucose or 300 kilocalories per day [50]. Treatment with SGLT2 inhibitors resulted in a reduction in body weight of 2.5 to 3 kg after 6 months, which was paralleled by a decrease in both the visceral and subcutaneous adipose tissue [44,45,51]. This weight loss reached a plateau notwithstanding the presence of persistent glucosuria. The lack of a further reduction in body weight can partially be attributed to an increase in energy intake [52].

### 2.3. Diuretic Effect of SGLT2 Inhibitors and Their Impact on Blood Pressure

As SGLT2 reabsorbs sodium and glucose at the same time, the administration of SGLT2 inhibitors also results in a natriuretic effect, which is most prominent in the first 2–3 days. Subsequently, natriuresis gradually returns to baseline levels over the course of several weeks. A median (interquartile range) plasma volume change from the baseline of −7.3% (−12.4% to −4.8%) was observed after 3 months of treatment [53]. Subsequent studies have revealed that the reduction in the plasma volume was maintained for 24 weeks [54,55]. In aggregate, these studies indicate that a new steady state of plasma volume is reached during chronic SGLT2 inhibition.

Blood pressure reduction by SGLT2 inhibitors is thought to be mediated by osmotic diuresis and mild natriuresis [56,57]. Moreover, the SGLT2 inhibitor-induced weight loss may also contribute to the reduction in blood pressure [58]. Additionally, the link with the renin-angiotensin-aldosterone (RAAS) system can also contribute to blood pressure reduction since inhibition of the reabsorption of sodium and glucose results in increased sodium delivery to the juxtaglomerular apparatus [59]. Consequently, the RAAS system is suppressed and the blood pressure is reduced [59]. The dapagliflozin blood pressure study showed that a dose of 10 mg resulted in a significantly more pronounced reduction in mean seated systolic blood pressure compared to the placebo group after 12 weeks (placebo-subtracted mean difference −4.28 mmHg, 95% CI −6.54 to −2.02; *p* = 0.0002) [60]. In the empagliflozin blood pressure study, the mean 24-h ambulatory measured systolic blood pressure was significantly lower in the empagliflozin 10 mg group (placebo-subtracted mean difference −3.44 mmHg; 95% CI −4.78 to −2.09; *p* < 0.001) and in the empagliflozin 25 mg group (placebo-subtracted mean difference −4.16 mmHg; 95% CI −5.5 to −2.83; *p* < 0.001) at week 12 [61].

### 2.4. Effects of SGLT2 Inhibitors on Atherosclerosis

Studies in experimental animal models of atherosclerosis have shown that SGLT2 inhibition attenuates the progression of atherosclerosis by a plenitude of mechanisms. These mechanisms include the inhibition of vascular inflammation, decreased oxidative stress, reversal of endothelial dysfunction, reduction in foam cell formation, and the prevention of platelet activation [62]. The majority of experimental atherosclerosis studies evaluating the impact of SGLT2 inhibitors have been performed in apolipoprotein E deficient mice with and without diabetes mellitus [63,64,65,66,67]. Not all studies have shown consistently beneficial effects. No effect on the atherosclerosis of dapagliflozin was observed in apo E deficient mice with heterozygous deficiency of insulin receptor substrate 2, which was characterized by insulin resistance [68].

### 2.5. Off-Target Effects and Adverse Effects of SGLT2 Inhibitors

SGLT2 inhibitors have also shown to exert effects that are independent of their interaction with SGLT2 receptors, which are termed off-target effects [69,70]. First, SGLT2 inhibitors depress the activity of Na^+^/H^+^ exchanger-3 located in the distal segment of the proximal tubule, contributing to natriuresis. Second, Na^+^-K^+^-2Cl^−^ cotransporters in the macula densa may also be inhibited by SGLT2 inhibitors, leading to the inhibition of glomerulotubular feedback. In addition, Na^+^/H^+^ exchanger-4, in the thick ascending limb of Henle, is also inhibited by SGLT2 inhibitors [69,70]. In the heart, SGLT2 inhibitors inhibit Na^+^/H^+^ exchanger-1 [71,72]. Moreover, Na^+^/H^+^ exchanger-11 is a potential target of SGLT2 inhibitors [73].

Adverse effects of SGLT2 inhibitors include the risk of genital mycotic infection and volume depletion-related events [74,75]. Dapagliflozin was reported to cause more diabetic ketoacidosis than the placebo group, while empagliflozin and canagliflozin were not [76,77,78].

In the Canagliflozin Cardiovascular Assessment Study (CANVAS), canagliflozin was shown to be associated with a higher risk of bone fracture and lower limb amputation [77]. However, in the later reported Canagliflozin and Renal Events in Diabetes with Established Nephropathy Clinical Evaluation (CREDENCE) trial [79], the fracture rates were not significantly different between the canagliflozin and placebo groups. There is no conclusive evidence that the intake of SGLT2 inhibitors is associated with an increased risk of cancer [80], although caution must be applied when treating bladder cancer patients with dapagliflozin, as shown by a pooled analysis of 22 clinical studies [81]. Data from large randomized clinical trials and real-world population-based studies have not demonstrated a significantly elevated risk of urinary tract infections in patients on SGLT2 inhibitors [82].

## 3. Cardiovascular Safety Studies on SGLT2 Inhibitors in Patients with Diabetes Mellitus

Type 2 diabetes mellitus is associated with an elevated risk of cardiovascular disease, and cardiovascular mortality is the primary cause of mortality in these patients [83,84]. Glucose-lowering drugs or strategies can be affiliated with an increased incidence of adverse cardiovascular outcomes [85]. Rosiglitazone, a peroxisome proliferator-activated receptor-γ agonist, is an antidiabetic drug of the thiazolidinedione class that improves insulin sensitivity. This compound has been demonstrated to increase the risk of cardiovascular events [86] and heart failure [87]. Moreover, the use of dipeptidyl peptidase-4 (DPP-4) inhibitors has been shown to result in an increased number of heart failure events in randomized clinical trials, and intake was associated with more heart failure events in observational studies [88]. The FDA mandated in 2008 and the EMA authorized in 2012 that clinical trials evaluating new anti-diabetic therapies should assess cardiovascular safety [21].

Since 2015, large-scale clinical trials have been launched to evaluate a potential impact of SGLT2 inhibitors on cardiovascular outcomes. In Table 1, the main four clinical trials are listed and their principal characteristics are compared. In general, these trials enrolled diabetic patients at high risk for cardiovascular events. The EMPA-REG OUTCOME and VERTIS-CV studies exclusively recruited patients with established cardiovascular disease, while the CANVAS and DECLARE-TIMI 58 trials extended the population to those with cardiovascular risk factors for cardiovascular disease. The 3-point major cardiovascular events (3P-MACE) composite endpoint was used as a primary composite endpoint in all listed studies, which consists of cardiovascular death, non-fatal myocardial infarction, and non-fatal stroke.

In the Empagliflozin Cardiovascular Outcome Event Trial in Type 2 Diabetes Mellitus Patients—Removal of Excess Glucose (EMPA-REG OUTCOME) trial [76], patients with type 2 diabetes and established cardiovascular disease receiving standard medical therapy (including lipid-lowering medication, antiplatelet drugs, and RAAS inhibitors) were randomized to receive empagliflozin (either 10 mg or 25 mg) or a placebo. After a median follow-up period of 3.1 years, the risk of 3P-MACE was 14% lower (hazard ratio (HR) 0.86, 95% CI 0.74–0.99, *p* < 0.001 for non-inferiority, *p* = 0.04 for superiority) in the empagliflozin group, mainly driven by a 38% reduced risk of cardiovascular death (HR 0.62, 95% CI 0.49–0.77, *p* < 0.001 for superiority). Hospitalization for heart failure was also significantly reduced by 35% with empagliflozin (HR 0.65, 95% CI 0.50–0.85, *p* = 0.002 for superiority), suggesting a potential for preventing heart failure hospitalization in diabetics. Additional subgroup analyses revealed a consistent effect of empagliflozin on cardiovascular mortality and hospitalization for heart failure regardless of the baseline risk of heart failure in type 2 diabetes [90].

The CANVAS study [77] assessed cardiovascular effects of canagliflozin (at a dose of either 100 mg or 300 mg) in patients with type 2 diabetes and at a high cardiovascular risk (i.e., either established cardiovascular disease or multiple cardiovascular risk factors). A 14% reduction in the risk of 3P-MACE was reached (HR 0.86, 95% CI 0.75–0.97, *p* < 0.001 for non-inferiority, *p* = 0.02 for superiority), while reductions in all-cause mortality and cardiovascular death were not significant. Hospitalization for heart failure as a secondary outcome was reduced by 33% (HR 0.67, 95% CI 0.52–0.87). Post hoc analysis of CANVAS showed no clear difference in the effects on HFrEF versus HFpEF events [91].

In the Dapagliflozin Effect on Cardiovascular Events—Thrombolysis in Myocardial Infarction 58 (DECLARE-TIMI 58) trial [78] in patients with type 2 diabetes and elevated cardiovascular risk, 3P-MACE was assessed as the primary safety and efficacy outcome. Additionally, the composite of cardiovascular death or hospitalization for heart failure was concurrently the co-primary efficacy outcome. Treatment with 10 mg dapagliflozin did not lead to a higher or lower rate of 3P-MACE compared to the placebo (HR 0.93; 95% CI 0.84–1.03; *p* < 0.001 for non-inferiority, *p* = 0.17 for superiority). The risk of cardiovascular death or hospitalization for heart failure was significantly lower in the dapagliflozin group (HR 0.83; 95% CI 0.73–0.95; *p* = 0.005 for superiority), mainly as a result of a great reduction in hospitalization for heart failure (HR 0.73, 95% CI 0.61–0.88), similar to that as observed in the EMPA-REG OUTCOME trial and the CANVAS trial.

The Evaluation of Ertugliflozin efficacy and safety cardiovascular outcomes trial (VERTIS-CV) [89] involving patients with type 2 diabetes and established cardiovascular disease demonstrated the non-inferiority of ertugliflozin but no significant reduction in 3P-MACE (HR 0.97, 95% CI 0.85–1.11; *p* < 0.001 for noninferiority), at a dose of either 5 mg or 15 mg. Further analyses demonstrated only a significant reduction in hospitalization for heart failure (HR 0.70, 95% CI 0.54–0.90).

In conclusion, the non-inferiority in relation to MACE indicated the cardiovascular safety of SGLT2 inhibitors in patients with type 2 diabetes with prior cardiovascular disease or at elevated risk for cardiovascular disease. Empagliflozin and canagliflozin have further shown cardiovascular benefits by significantly reducing the risk of MACE. Notably, all four mentioned SGLT2 inhibitors have demonstrated potential in decreasing hospitalization for heart failure in diabetic patients, which has motivated further research into SGLT2 inhibitors in heart failure patients. Furthermore, studies were extended to patients without type 2 diabetes.

Nevertheless, hospitalization for heart failure is only one particular clinical outcome endpoint in the treatment of chronic heart failure. Indeed, the treatment of heart failure is directed toward different goals. In order to make a comprehensive interpretation of the currently available evidence regarding the effects of SGLT2 inhibitors in heart failure, the therapeutic goals of heart failure treatment need to be accurately defined. Heart failure endpoints need to be precisely circumscribed and the rationale behind composite endpoints needs to be explored. These topics are discussed in depth in Section 4 and applied in Section 5.

## 4. Definition of Heart Failure Endpoints

Clinical endpoint adjudication requires unambiguous definitions. Objective criteria have been developed, contributing to the standardization of definitions [92,93,94,95]. In general, endpoints in studies of heart failure are defined according to the therapeutic goals of heart failure management. The major goals of heart failure treatment are described in the most recent European Society of Cardiology (ESC) guidelines for HFrEF [4]: (1) reduction in (cardiovascular) mortality, (2) prevention of recurrent hospitalizations due to worsening heart failure, and (3) improvement in clinical status, functional capacity, and quality of life. The third goal can be comprehensively described as the evaluation of the impact of an intervention on heart failure-related health status, but this health status cannot be summarized by one single metric. Ideally, an effective treatment should achieve all three goals. However, the impact of interventions on these three different goals are not always concordant.

### 4.1. All-Cause Mortality and Cardiovascular Mortality

The progress of heart failure management has been partially based on the development of deeper insights into the pathophysiology of heart failure and the concomitant development of new paradigms. Since William Withering first described heart failure as “dropsy” in the 18th century, it had been regarded as an edematous disease until 1960s. Diuretics were accordingly used as the main treatment to stimulate fluid excretion via urine [96]. However, clinical observations and hemodynamic data demonstrated the chronic character of heart failure, as reflected by the persistent hemodynamic abnormalities and persistent limitations in exercise capacity, even after the edema is relieved [97]. In the 1980s and early 1990s, evidence showed that elevated plasma levels of neurohormones (e.g., norepinephrine and renin) in heart failure patients were associated with worse long-term prognosis and reduced survival. Clinical trials established that β-blockers and angiotensin-converting enzyme inhibitors led to improved survival [98,99]. Reducing mortality and improving survival therefore became an important objective in the treatment of heart failure, along with relieving symptoms. Treatments that can alter the natural history of heart failure and modify the risk of mortality are labeled as disease-modifying therapies in contrast to interventions that only have beneficial effects on symptoms.

The Cooperative North Scandinavian Enalapril Survival Study (CONSENSUS) in 1987 was a landmark trial that assessed all-cause mortality as the primary endpoint [100]. Patients with severe congestive heart failure (New York Heart Association (NYHA) functional class IV) treated with the angiotensin-converting enzyme inhibitor enalapril demonstrated a reduced total mortality and an improvement in symptoms after 6 and 12 months [100]. Regarding the causes of mortality, cardiovascular mortality accounted for more than 99% of deaths (117 of 118 deaths). This is a clear-cut and exceptional result. Nevertheless, this result is contingent on the advanced heart failure profile of participants and contingent on the fact that the placebo group was not treated with other disease-modifying therapies that were proven to be efficacious in the subsequent two decades.

All-cause mortality is the most clear-cut endpoint. Cardiovascular mortality includes deaths that result from an acute myocardial infarction, sudden cardiac death, death due to heart failure, death due to stroke, death due to cardiovascular procedures, death due to cardiovascular hemorrhage, and death due to other cardiovascular causes [92]. Since one assumes that heart failure treatments affect cardiovascular mortality and are rather neutral in relation to non-cardiovascular deaths, cardiovascular mortality has also been used as a primary efficacy outcome. However, it is still indicated to assess total mortality as a safety endpoint to ensure that survival is not adversely affected by unexpected mechanisms, since some treatments for heart failure could increase the risk of death [101,102,103,104]. Using cause-specific endpoints such as cardiovascular mortality requires a critical definition and adjudication of specific events. Differences in definition and/or adjudication of events can lead to heterogeneity among clinical trials [92].

### 4.2. Worsening Heart Failure Events

When the incidence of death or cardiovascular death is not high, the execution of clinical trials with all-cause mortality or cardiovascular mortality as the endpoint becomes problematic from the perspective of statistical power. As the rate of mortality in chronic heart failure appears to be declining [9] and is also lower in patients with less severe heart failure, conducting large-scale trials with (cardiovascular) mortality as the primary endpoint have become impractical due to the very large sample size that would be required to generate a sufficient number of events corresponding to the primary endpoint. Furthermore, mortality does not completely capture the clinical condition of patients during the treatment period. One patient with an objective and stable clinical improvement and a second patient with severe and persistent symptoms leading to repeated hospitalization who both survive until the end would not be distinguished in the clinical trial primary endpoint. Worsening heart failure events are episodes of worsening signs or symptoms despite ongoing therapy that require hospitalization or outpatient escalation of therapy [95,105]. Heart failure hospitalization is defined as an unscheduled hospital admission with heart failure as the primary diagnosis that lasts longer than 24 h or crosses a calendar day [95]. A non-hospitalized heart failure event is defined as an urgent, unscheduled office or emergency visit for heart failure [95].

### 4.3. Composite Endpoints of Fatal and Nonfatal Events

Composite endpoints, the combination of two or more study outcomes into a single composite measure, have been increasingly used in the past decades as a primary efficacy measure in heart failure trials [106]. The advantage of composite endpoints is that the higher number of events increases the precision of the estimates and allows for the execution of clinical trials with a smaller number of subjects and/or a shorter duration of follow-up [107]. Another advantage is that composite endpoints comprising both fatal and non-fatal events avoid the problem of competing risks [106,108]. However, the correct use of a composite endpoint is dependent on a series of conditions. In this respect, Montori et al. [109] proposed criteria for the validity of composite endpoints in 2005. The first criterion is that the endpoint components must be of similar importance to patients. Second, the incidence rate of the components must be alike, otherwise the effect on the composite outcome may be determined by the most predominant event. Third, the effect of the treatment should be similar for each component of the composite endpoint. The composite endpoint is substantively and logically coherent when the underlying biology and pathophysiology of the different components is similar enough so that risk reductions of the same magnitude can be expected [110]. Discordant results in individual components of a primary composite endpoint are concerning [106] and clearly hamper the interpretation of clinical trial results. A key problem with composite endpoints is that non-fatal events and fatal events have equal statistical weighting and this matter is further discussed in Section 7.1.

A typical primary composite endpoint in heart failure trials is the composite of cardiovascular mortality and heart failure hospitalization, which is regarded as a cause-specific composite endpoint. Since the beginning of the 21st century, most studies of disease-modifying treatments for chronic heart failure have indeed employed the composite endpoint of cardiovascular mortality and hospitalization for heart failure [111,112,113,114,115,116,117,118] as the primary endpoint, while in earlier trials, all-cause mortality was generally assessed [119,120,121]. The scientific argument for such a combined endpoint is originally based on the Studies of Left Ventricular Dysfunction (SOLVD) [122]. The SOLVD study, published 4 years later than the CONSENSUS study, focused on chronic heart failure patients with a left ventricle ejection fraction equal or less than 35% from all NYHA functional classes (approximately 90% NYHA II and III) and extended the treatment period to 41.4 months. All-cause mortality and cardiovascular mortality were both significantly lower in the enalapril group. Notably, there was no difference in the number of deaths between the enalapril and the placebo group among those who were not hospitalized during the trial for worsening heart failure, which indicates that the significant difference in mortality in this trial selectively occurred in patients who had been hospitalized for heart failure. Hospital admission is indeed a sentinel event in the course of chronic heart failure with hospitalized patients having mortality rates that are substantially increased compared to never hospitalized patients [123,124,125,126]. However, it is important to consider the nature of the association between heart failure hospitalization and mortality. The presence of an independent relationship between prior hospitalization and mortality after comprehensive risk adjustment for clinical confounders would be compatible with the organ injury hypothesis of heart failure hospitalization, implying that heart failure hospitalization causes an irreversible inflection point in the natural history [123]. In an analysis of the ASCEND-HF trial (Acute Study of Clinical Effectiveness of Nesiritide in Decompensated Heart Failure) cohort, with stepwise adjustment for patient factors measured at the study baseline, no independent association between heart failure hospitalization and subsequent mortality was observed [123]. This indicates that hospitalization for worsening heart failure represents a risk marker of sicker patients with chronic heart failure but is not an independent risk factor for mortality. In any case, the assumption that a reduction in heart failure hospitalization will translate in a subsequent reduced (cardiovascular) mortality risk may not be consistently valid. This is not only theoretical but has been demonstrated in several clinical trials [114,127]. In the SHIFT trial (Systolic Heart Failure Treatment with the I_f_ Inhibitor Ivabradine Trial), no significant effect of ivabradine was observed on cardiovascular mortality whereas a significant impact of ivabradine on the primary composite endpoint of cardiovascular death or hospital admission for worsening heart failure was demonstrated (HR 0.82, 95% CI 0.75–0.90; *p* < 0.0001) [114]. Similarly, in the DIG (Digitalis Investigation Group) trial, no effect was observed of digoxin on mortality or on cardiovascular mortality whereas fewer patients were hospitalized for worsening heart failure in the digoxin group than in the placebo group (risk ratio 0.72, 95% CI 0.66 to 0.79; *p* < 0.001) [127].

### 4.4. Clinical Status, Functional Capacity, and Quality of Life

In chronic conditions such as heart failure, hard clinical endpoints like all-cause mortality or cardiovascular mortality and a softer endpoint like hospitalization for worsening heart failure are not the only meaningful efficacy measure. Patients who are alive and not hospitalized can still have a poor clinical status, functional capacity, or quality of life [128]. From the perspective of patients, the heart failure-related health status is extremely meaningful. A clinically meaningful improvement by therapy can be experienced by an individual patient whereas the impact on mortality cannot be demonstrated at the level of the individual, only at the level of the population. Some patients may even prefer an improved quality of life and functionality over an effect on mortality [129,130]. The challenge is, however, to develop adequate metrics of heart failure-related health status and to interpret these metrics in terms of what is clinically meaningful.

The evaluation of heart failure-related health status was assessed by means of short-to-intermediate-term studies. In contrast to long-term trials focusing on mortality and hospitalization, these trials focusing on symptoms and clinical status were frequently inconsistent [131]. Measures that were used in studies included: the direct assessment of symptoms, exercise tolerance, NYHA functional class, global assessment of progress, and quality of life assessments [132].

Several approaches for the evaluation of clinical status have been applied. The direct assessment of symptoms examines the presence and severity of specific symptoms (e.g., dyspnea or fatigue) during specified levels of effort (e.g., at rest and during exertion) [132]. The NYHA functional class combines subjective assessments of the patient’s clinical status and assigns the patient to one of several classes, according to the degree of effort needed to elicit symptoms [132]. Similarly, the global assessment of progress also uses subjective judgments to detect the direction and magnitude of the change in the patient’s clinical status [132]. In addition, exercise tolerance is measured through exercise testing to assess the ability of the treatment to prolong exercise. For instance, the 6-min walk test measuring the walking distance within 6 min was shown to be strongly and independently associated with mortality and hospitalization for heart failure in patients with left ventricular dysfunction [133]. Finally, quality of life assessments have been developed to detect the range of physical, emotional, functional, and cognitive impairments via questionnaires, which may be general or disease specific [132]. The Kansas City Cardiomyopathy Questionnaire (KCCQ) is the most commonly used and validated heart failure disease-specific instrument. It consists of 23 items exploring six domains: symptoms, physical function, quality of life, social limitation, self-efficacy, and symptom stability [134]. Domains are scored on a scale from 0 to 100, with a higher score indicating fewer symptoms and a change of 5 or more points considered to be clinically meaningful [135]. The combination of the functional status with the quality of life and social limitation domains forms a clinical summary score [135].

## 5. Effect of SGLT2 Inhibitors in Chronic Heart Failure

The cardiovascular trials of SGLT2 inhibitors in type 2 diabetes discussed in Section 3 have generally adopted MACE as the primary safety and/or efficacy endpoint, which tends to emphasize ischemic endpoints but not heart failure events. Furthermore, the study population in these trials was limited exclusively to diabetic patients and was very distinct compared to patients with prevalent heart failure. Nevertheless, the data of these original safety trials have been the impetus and stimulus to conduct new trials evaluating the impact of SGLT2 inhibitors on heart failure outcomes in patients with established heart failure, both with diabetes mellitus and without diabetes mellitus. Heart failure patients with a left ventricle ejection fraction less than 40% (HFrEF) and greater than 40% were separately analyzed. A post hoc analysis [136] of dapagliflozin in patients with type 2 diabetes mellitus [78] provided an interesting perspective. Of the 17,160 patients included in this trial, 671 (3.9%) had HFrEF at baseline, 1316 (7.7%) had heart failure without known reduced EF, and 15,173 (88.4%) had no history of heart failure at baseline. Dapagliflozin diminished heart failure hospitalization in patients with HFrEF (HR 0.64, 95% CI 0.43–0.95) and without HFrEF (HR 0.76, 95% CI 0.62–0.92) and reduced cardiovascular deaths (HR 0.55, 95% CI 0.34–0.90) and all-cause mortality (HR 0.59, 95% CI 0.40–0.88) exclusively in patients with HFrEF [136]. This analysis suggest that a composite endpoint of cardiovascular death and heart failure hospitalization may be cumbersome and problematic in heart failure patients without HFrEF. These data suggest that the impact of SGLT2 inhibitors in heart failure patients may be dependent on the ejection fraction.

In Table 2, four large-scale trials evaluating the cardiovascular outcomes of SGLT2 inhibitors in heart failure are listed and compared.

### 5.1. HFrEF

The DAPA-HF (The Dapagliflozin and Prevention of Adverse Outcomes in Heart Failure) trial in 2019 investigated the efficacy of dapagliflozin in patients with established heart failure and a reduced ejection fraction (ejection fraction of 40% or less) regardless of type 2 diabetes mellitus status [117]. The risk of the primary composite outcome (i.e., cardiovascular death or worsening heart failure) was reduced with dapagliflozin as an add-on therapy (HR 0.74, 95% CI 0.65–0.85, *p* < 0.001) (Table 2). Worsening heart failure as one component of the composite refers to hospitalization for heart failure or urgent medical visit resulting in intravenous therapy for heart failure (Table 2). Separate analysis on worsening heart failure was also solely shown to occur less frequently in the dapagliflozin group (Table 2). This study is the only clinical trial to date that has shown that an SGLT2 inhibitor resulted in a significant reduction in cardiovascular mortality in heart failure patients. The result is consistent with the post hoc analysis of DECLARE-TIMI 58 described above [136].

The EMPEROR-Reduced (Empagliflozin Outcome Trial in Patients with Chronic Heart Failure with Reduced Ejection Fraction) trial, published shortly after the DAPA-HF trial, assessed the cardiovascular efficacy of empagliflozin in HFrEF patients (ejection fraction of 40% or less). Administrating empagliflozin 10 mg once daily on top of the standard heart failure therapies efficaciously lowered the risk of cardiovascular death or hospitalization for heart failure (HR 0.75, 95% CI 0.65–0.86, *p* < 0.001), regardless of the presence or absence of diabetes [118]. In contrast to the DAPA-HF trial, cardiovascular mortality was not significantly reduced with empagliflozin in this study and there was also no clear or strong trend, implying that the impact of the drug on the primary endpoint was mainly driven by hospitalizations for heart failure (Table 2).

Based on the findings from the DAPA-HF and EMPEROR-Reduced studies, dapagliflozin and empagliflozin are strongly recommended in current clinical practice guidelines as a treatment in patients with HFrEF (NYHA class II-IV heart failure) [4,139]. However, based on the data of both trials, it cannot be excluded that both drugs have dissimilar effects on cardiovascular mortality in HFrEF patients.

In addition, both trials also assessed the functional outcomes by means of the KCCQ, but they evaluated different domains of KCCQ and the duration of the observation period was also distinct. In the DAPA-HF study, the KCCQ total symptom score was measured. The use of dapagliflozin resulted in a greater increase in the total symptom score on the KCCQ at month 8 compared to the baseline (win ratio 1.18; 95% CI 1.11–1.26; *p* < 0.001), indicating its potential in ameliorating symptoms among heart failure patients [117]. However, while this is a statistically significant effect, the impact is therefore not necessarily clinically meaningful. The EMPEROR-Reduced trial also evaluated the functional impact of empagliflozin by determining the change in the quality-of-life score on KCCQ at 52 weeks, which was not significantly different between the empagliflozin and the placebo groups [118].

The CHIEF-HF (Canagliflozin: Impact on Health Status, Quality of Life and Functional Status in Heart Failure) was patient centered and conducted in a completely remote fashion [140]. This trial evaluated the health status benefits of canagliflozin [140] in patients with HFrEF and HFpEF by employing change in the KCCQ total symptom score as its primary outcome. The study drug was distributed remotely and the data were collected through smartphones. The mean difference in the changes in scores at 12 weeks was 4.3 points (95% CI 0.8–7.8; *p* = 0.016) in favor of canagliflozin, demonstrating a statistically significant amelioration in symptom burden in patients with heart failure of all types after a 12-week treatment of canagliflozin. HFrEF patients accounted for 40.4% of the study population, in which the mean difference in the changes of scores was 4.0 points (95% CI −1.0–9.0).

Collectively, these data indicate that dapagliflozin and canagliflozin but not empagliflozin had a statistically significant effect on heart failure-related health status in HFrEF patients. Whether the observed impact is clinically meaningful is a matter of debate. Given the importance of symptoms, function, and quality of life to patients, more clinical trials assessing the functional impact of SGLT2 inhibitors are ongoing. The DETERMINE-Reduced [141] and the EMPERIAL-Reduced [142] will evaluate dapagliflozin and empagliflozin, respectively, in HFrEF patients by means of the KCCQ and 6-min walking distance.

### 5.2. HFmrEF and HFpEF

The EMPEROR-Preserved (Empagliflozin Outcome Trial in Patients with Chronic Heart Failure with Preserved Ejection Fraction) trial was carried out to evaluate the effects of empagliflozin on major heart failure outcomes in heart failure patients with an ejection fraction greater than 40% [137]. SGLT2 inhibition with empagliflozin reduced the combined risk of cardiovascular death or hospitalization for heart failure by 21% (HR 0.79, 95% CI 0.69–0.90, *p* < 0.001), which was independent of the presence or absence of diabetes. The effect on the primary endpoint was mainly driven by a 29% lower risk of hospitalization for heart failure (HR 0.71, 95% CI 0.60–0.83). The risk of cardiovascular death was not significantly different between the empagliflozin and placebo groups (HR 0.91, 95% CI 0.76–1.09). Subgroup analyses suggested that patients with an ejection fraction of 40% to 49% benefited most from the cardiovascular effects of empagliflozin. The primary outcome reduction was not significant in the group with an ejection fraction of 60% or more.

Recently, the DELIVER (Dapagliflozin Evaluation to Improve the Lives of Patients with Preserved Ejection Fraction Heart Failure) trial revealed the cardiovascular effects of dapagliflozin in patients with HFmrEF (ejection fraction between 41% and 49%) or HFpEF (ejection fraction of 50% or more) [138]. Dapagliflozin resulted in a lower risk in the primary composite outcome of worsening heart failure and cardiovascular death (HR 0.82, 95% CI 0.73–0.92, *p* < 0.001), without a difference in benefit among patients with an ejection fraction of 60% or more and those with an ejection fraction of less than 60%. The effect of dapagliflozin on the primary outcome was consistent in subgroups with or without type 2 diabetes. There was no significant effect of dapagliflozin on cardiovascular mortality. Collectively, the reduction in the primary composite endpoint in the two HFmrEF and HFpEF patients was mainly driven by a markedly lower risk of hospitalization for heart failure, while cardiovascular deaths in both trials were not substantially reduced in the SGLT2 inhibitor treatment group.

A prespecified analysis of the DELIVER trial examined the efficacy of dapagliflozin in the group of heart failure patients with improved ejection fraction, defined as patients whose ejection fraction improved from 40% or less to more than 40% [143]. This population has a high event rate and were therefore generally excluded from other trials. In the DELIVER trial, this group of patients with improved ejection fraction was included and accounted for 18% of the study population. Dapagliflozin showed consistent effects on the primary composite outcome in this group (HR 0.74, 95% CI 0.56–0.97). In particular, the cardiovascular deaths in this group were also significantly reduced (HR 0.62, 95% CI 0.41–0.96) in contrast to the general study population [143]. Another prespecified analysis of the DELIVER trial showed that treatment with dapagliflozin provided a clinical benefit irrespective of the baseline NYHA class [144]. Dapagliflozin improved the NYHA class as early as 4 weeks [144].

A meta-analysis of the DELIVER and EMPEROR-preserved trials was prespecified in the DELIVER academic statistical analysis plan and preregistered with PROSPERO (CRD42022327527) before unmasking of the DELIVER trial results [145]. Importantly, no statistically significant effect on cardiovascular mortality was observed in this meta-analysis of both trials. When the total mortality was analyzed, there was no trend showing a difference (HR 0.97, 95% CI 0.88–1.06). In contrast, a prespecified meta-analysis of the DAPA-HF and the EMPEROR-Reduced trial showed a significant effect on cardiovascular mortality (HR 0.86, 95% CI 0.76–0.98; *p* = 0.027) and on all-cause mortality (HR 0.87, 95% CI 0.77–0.98; *p* = 0.018) among the 8474 patients with HFrEF [146].

Similarly, as in the HFrEF trials, studies on HFmrEF and HFpEF patients also carried out more detailed evaluations of heart failure-related health status. In the EMPEROR-Preserved, no significant improvement in the quality-of-life score on KCCQ at 52 weeks was observed [137]. The DELIVER trial showed a minor decrease in symptom burden with dapagliflozin, as measured by an increase in the KCCQ total symptom score at month 8 (win ratio 1.11; 95% CI 1.03–1.21; *p* = 0.009; mean placebo-corrected difference between baseline and month 8 among survivors 2.4 points; 95% CI 1.5–3.4) [138]. Dapagliflozin resulted in a statistically significant improvement in the exercise capacity of HFpEF patients (defined as with an ejection fraction of 45% or more) in another trial including 324 patients [147]. The KCCQ clinical summary score, evaluated as the primary endpoint (effect size 5.8 points; 95% CI 2.3–9.2, *p* = 0.001), was improved, together with an amelioration of the 6-min walk test (mean effect size of 20.1 m; 95% CI 5.6–34.7, *p* = 0.007) after a 12-week dapagliflozin treatment [147]. In the CHIEF-HF trial, the mean difference in the changes in scores was 4.5 points (95% CI −0.3–9.4) in the HFpEF patients, which was similar to that of the HFrEF patients (*p* value for interaction = 0.35) [140]. The ongoing DETERMINE-Preserved [148] and EMPERIAL-Preserved [142] trials will assess the functional impact of dapagliflozin and empagliflozin, respectively, making use of the KCCQ and 6-min walking distance.

## 6. Evaluation of the Interchangeability of SGLT2 Inhibitors in Treatment of Chronic Heart Failure

Therapeutic classes are defined based on the mechanism of action. Drugs of a particular class share a single mechanism, but at the same time, all drugs have multiple actions that may be favorable and unfavorable. The more similarities between the agents within a pharmacologic class in terms of chemical structure, pharmacodynamics, and pharmacokinetics, the greater the likelihood of shared class effects [149]. In the HFrEF trials, a significant effect of dapagliflozin on cardiovascular mortality was observed in the DAPA-HF trial, whereas no significant effect was observed for empagliflozin on cardiovascular mortality in the EMPEROR-Reduced trial [117,118]. Differences between different drugs in the same class in clinical trials may be due to chance, methodological heterogeneity, clinical heterogeneity (effect-measure modification related to clinical characteristics of patients included in trials), and finally, may be caused by differences in the unique benefits or harm of individual drugs of the same class.

### 6.1. Methodological Heterogeneity in Heart Failure Endpoints Definition

There is a certain degree of heterogeneity in the definitions of endpoints in the existing studies, impeding a comparison of the current evidence.

Meaningful differences in the defining components of composite endpoints exist between the DAPA-HF and EMPEROR-Reduced trials [117,118]. The primary composite outcome of DAPA-HF was cardiovascular death or worsening heart failure. Worsening heart failure in DAPA-HF included unplanned hospitalizations or urgent visits with intravenous therapy. In contrast, the EMPEROR-Reduced trial used cardiovascular death or hospitalization for heart failure as the primary endpoint, excluding urgent heart failure visits. In relation to hospitalization for heart failure, DAPA-HF incorporated hospitalization for heart failure with a length-of-stay exceeding 24 h whereas EMPEROR-Reduced also included events of 12 to 24 h if intervention was not restricted to oral diuretics. Regarding cardiovascular deaths, DAPA-HF excluded undetermined causes of death from the primary endpoint and this was distinct in the EMPEROR-Reduced. When non-differential misclassification of outcomes occurs, this will attenuate an effect on cardiovascular mortality in the EMPEROR-Reduced. Therefore, non-differential misclassification may, in theory, partially explain why the risk of cardiovascular death in DAPA-HF was significantly lower in the dapagliflozin group whereas in the EMPEROR-Reduced trial, no significant effect on cardiovascular mortality was observed. On the other hand, it is unlikely that differential outcome misclassification can occur within a randomized trial in which the outcome assessor is blinded to treatment allocation.

Regarding the evaluation of heart failure-related health status, both HFrEF trials used KCCQ as the endpoint, but DAPA-HF employed the change in the total symptom score on KCCQ from the baseline to 8 months whereas EMPEROR-Reduced chose to use change in a clinical summary score on KCCQ from the baseline to 52 weeks. DAPA-HF measured a statistically substantial improvement in the score in the dapagliflozin group, in contrast to the EMPEROR-Reduced where no significant difference between the empagliflozin group and the placebo group was observed. This discordance in functional benefits between empagliflozin and dapagliflozin can potentially indicate drug-specific effects rather than class-specific effects, although the difference in duration of observation might also play a role. Nevertheless, the real issue here is that it is questionable whether a clinically relevant benefit on KCCQ was observed in any of these trials.

The EMPEROR-Preserved and DELIVER trials on HFmrEF and HFpEF patients have generally adopted the definition of primary endpoints from the EMPEROR-Reduced and DAPA-HF trials, respectively [137,138]. Post hoc analysis applied DELIVER endpoint definitions on EMPEROR-Preserved data, showing a modest impact on the effect size [150]. Using the endpoint definitions from DELIVER (urgent heart failure visits were added and undetermined death as part of cardiovascular death was eliminated from the primary composite endpoint), the primary outcome overall occurred in 13.1% in the empagliflozin and 16.8% in the placebo groups (HR 0.76, 95% CI 0.67–0.87; *p* < 0.0001) [150]. However, this matter tends to obscure the real problem. The effect on the primary endpoint in both trials is mainly driven by worsening heart failure events. The real issue is that it is highly unlikely that there is a clinically meaningful effect of SGLT2 inhibitors on cardiovascular mortality in HFpEF trials.

### 6.2. Clinical Heterogeneity in SGLT2 Inhibitor Chronic Heart Failure Trials

#### 6.2.1. HFrEF

In the two major outcome trials of dapagliflozin and empagliflozin on HFrEF patients, cardiovascular death as one component of the primary composite outcome was only significantly reduced in the DAPA-HF trial. Specifically, the risk reduction in cardiovascular mortality was 18% (HR 0.82, 95% CI 0.69–0.98) in DAPA-HF [117] and a non-significant 8% (HR 0.92, 0.75–1.12) in EMPEROR-Reduced [118]. In contrast, in earlier trials on patients with type 2 diabetes at high cardiovascular risk, the reduction in cardiovascular mortality was absent (HR 0.98, 95% CI 0.82–1.17) in DECLARE-TIMI 58 assessing dapagliflozin [78] and 38% (HR 0.62, 95% CI 0.49–0.77) in EMPA-REG OUTCOME assessing empagliflozin [76]. The patient characteristics in the cardiovascular safety trials and in the heart failure trials were highly distinct. A second consideration is that the absolute number of cardiovascular deaths in these trials was highly distinct between these trials. In DAPA-HF, 227 cardiovascular deaths (9.6%) were observed in the dapagliflozin group compared to 273 cardiovascular deaths (11.5%) in the placebo group [117]. In EMPEROR-Reduced, the number of cardiovascular deaths were 187 (10.0%) and 202 (10.8%) in the empagliflozin group and the placebo group, respectively [118]. In DECLARE-TIMI 58, 245 (2.9%) and 249 (2.9%) cardiovascular deaths were measured in the dapagliflozin and placebo groups, respectively [78]. In the EMPA-REG OUTCOME, there were 172 (3.7%) cardiovascular deaths in the empagliflozin group compared with 137 (5.9%) in the placebo group [76].

Regarding the profile of the study population, the DAPA-HF trial primarily enrolled patients with mild-to-moderate degrees of left ventricular systolic dysfunction [117], while the EMPEROR-Reduced was enriched for patients with a greater severity of left ventricular systolic dysfunction [118]. Compared with those in DAPA-HF, patients enrolled in the EMPEROR-Reduced trial had a lower ejection fraction (27% vs. 31%).

#### 6.2.2. HFmrEF and HFpEF

As mentioned before, the subgroup analyses in EMPEROR-Preserved showed that the primary outcome was not significantly reduced in the group with the ejection fraction of 60% or more [137]. Furthermore, a post hoc analysis of EMPEROR-Reduced and EMPEROR-Preserved, in which patients were grouped based on ejection fraction, revealed a potential attenuation of the benefit of empagliflozin in patients with a LVEF ≥ 65% [151]. DELIVER has therefore carried out dual primary analyses in patients with a LVEF of less than 60%, in addition to the overall patient population, to detect a potential decrease in effect as the LVEF increases [138]. In contrast to the EMPEROR trials, no difference in benefit was observed among patients with a LVEF of more or less than 60%, indicating that the benefit of dapagliflozin is likely to extend throughout the full range of ejection fraction.

### 6.3. Class-Specific Effects of SGLT2 Inhibitors

Based on the currently available clinical trial data, the effects on clinical endpoints in chronic heart failure data represent predominantly class-specific effects rather than drug-specific effects. It cannot entirely be excluded that minor differences between individual drug members of the class exist based on the data presented in Section 6.2.2 and based on the distinct impact of dapagliflozin and empagliflozin on KCCQ as the endpoint.

## 7. Perspectives, Future Directions, and Conclusions

### 7.1. Hierarchical Composite Endpoints and Win Ratio

The application of SGLT2 inhibitors in the treatment of acute heart failure was outside the focus of this review. A recent review concluded that currently available studies including six randomized trials and two real-world studies provide conflicting results concerning the true efficacy of SGLT 2 inhibitors in the setting of acute heart failure [152]. Interestingly, the win ratio was used to evaluate the impact of empagliflozin on the primary composite endpoint in the EMPULSE (Empagliflozin in Patients Hospitalized With Acute Heart Failure Who Have Been Stabilized) acute heart failure trial [153]. This introduces an additional dimension in the complexity of the interpretation of primary endpoints. In 2013, Pocock and colleagues [154] proposed the win ratio as a new method for reporting composite endpoints, which appropriates priority to the more clinically important event (e.g. mortality), and is a generalized pairwise comparison technique [155]. In a first step, endpoints are prioritized, typically by clinical severity, and this generates a hierarchical order. Subsequently, all possible pairs are constructed consisting of one patient from each group. In each of these pairs, the comparison starts with the most clinically severe endpoint, and the subject with the better outcome (a ‘win’) is identified. If there is no win, the process is repeated for the next component and further repeated until a win is declared. If there is finally no win in the component with the least priority, a tie is declared. The advantage is that priority is given to more important components. The win ratio is the total number of winners divided by the total numbers of losers. In an analysis of 16 large cardiovascular outcome trials, the hazard ratios and win ratios provided similar estimates of treatment effects [156]. In other words, the results of ‘time-to-worst event analysis’ (hierarchy of the components) was similar compared to the ‘time-to-event’ analysis (no hierarchy of components). However, the win ratio also allows for the incorporation of patient-centered and other outcomes in the primary endpoint while prioritizing the competing risk of death and hospital admission [156]. This has been applied in the EMPULSE trial [153], where 530 patients with a primary diagnosis of acute de novo or decompensated chronic heart failure regardless of left ventricular ejection fraction were randomly assigned to receive empagliflozin 10 mg once daily or the placebo. The primary outcome of the trial was clinical benefit, defined as a hierarchical composite of death from any cause, number of heart failure events, and time to first heart failure event, or a 5 point or greater difference in change from the baseline in the KCCQ Total Symptom Score at 90 days [153]. The stratified win ratio was 1.36 favoring empagliflozin (95% CI 1.09–1.68; *p* = 0.0054). The interpretation of clinical trial results with hierarchical composite endpoints will become more complex since it will be less clear what drives a statistical difference. Very disparate categories are integrated in one composite endpoint. When the mortality assessment time window is short, the influence or contribution of mortality on the composite is diminished relative to a patient-centered outcome like dyspnea. Detailed criticisms have been provided by Brown and Ezekowitz [157].

Hierarchical clinical composite endpoints certainly do not solve the main problems associated with classical composite clinical endpoints. Both strategies merge event types, which lead to a loss of information, and both do not provide event-specific estimates of the effect [158]. Brown and Ezekowitz [158] proposed incorporating all recurrent events in the analysis and advanced a more sophisticated approach that they described as multitype recurrent events (MTREs) analysis [158]. Whether this sophisticated approach will become widely applied is not clear at present. In contrast, the win ratio is increasingly adopted within the cardiovascular field [155] and will pose further challenges to the interpretation of trial results.

### 7.2. Real-World Effectiveness versus Clinical Trial Efficacy

In general, patients that are included in clinical trials are not entirely representative of patients in clinical practice. Although many other reasons may contribute to the infrequent prescription of certain classes of heart failure drugs in the real world [159], differences between heart failure patients that meet the inclusion criteria of clinical trials and are willing to participate in clinical trials on the one hand, and the general heart failure population on the other hand, should also be considered. Clinical register data offer the possibility of evaluating the effectiveness of SGLT2 inhibitors in the real world. Although these data are observational and problems of confounding by indication may occur, conditioning on a propensity score can be applied to balance measured covariables.

### 7.3. Conclusions

In this review, the progress from SGLT2 inhibitors being evaluated as anti-diabetic agents to SGLT2 inhibitors being assessed as a therapy for chronic heart failure was illustrated. SGLT2 inhibitors with glucose-lowering effects independent of augmented insulin secretion have shown cardiovascular benefits in cardiovascular safety studies in patients with type 2 diabetes mellitus. The positive effect on hospitalization for heart failure stimulated further research into the effects of SGLT2 inhibitors on heart failure outcomes. This was realized by large-scale clinical trials performed in different categories of heart failure patients, with defined endpoints compiled in line with the therapeutic goals of heart failure management. SGLT2 inhibitors exerted a statistically significant effect on the primary outcome in both the HFrEF and HFpEF trials. The effect on hospitalization for heart failure was unequivocal, whereas the effect on cardiovascular mortality was both statistically and clinically insignificant with the exception of the DAPA-HF trial. The composite endpoint of cardiovascular death and hospitalization of heart failure/worsening heart failure was used as the primary endpoint in the clinical trials. This was not justified in both HFpEF trials, and this field illustrates the problem of using composite endpoints when the effect is driven by less serious components. The only consistent result is an effect on heart failure hospitalization, and this component should not have been combined with cardiovascular mortality.

In addition to evaluating the first two therapeutic goals of heart failure treatment being reducing mortality and preventing hospitalization for heart failure, the third goal concerning the heart failure-related health status was also assessed by means of the KCCQ. To date, heart failure-related health status has mainly been analyzed as a secondary outcome; more large-scale trials assessing functionality and health status as the primary outcome endpoint are ongoing. Up until now, there have been no convincing data that there is a clinically meaningful improvement in the heart failure-related health status of these patients. Statistically significant improvements in certain metrics do not necessarily indicate a clinically meaningful effect.

Whether there is or is not a clinically meaningful impact of SGLT2 inhibitors on heart failure-related health status is further being evaluated in ongoing trials [141,142,148]. From the perspective of the patient, such an improvement is what is most meaningful. Unlike the primary and secondary prevention of ischemic cardiovascular events, treatment of heart failure is not uniquely or necessarily (fatal or non-fatal) event-driven but can also be driven by the clinical status of the patient. An impact on the heart failure-related health status can make an intervention attractive, even in the absence of an effect on major events. Ongoing trials [141,142,148] primarily assessing the heart failure-related health status are of great importance, especially since existing trials clearly suffered from the limitations of the composite endpoint of mortality and hospitalization for heart failure.

## Figures and Tables

**Table 1:** Large-scale clinical trials assessing cardiovascular safety of SGLT2 inhibitors.

	EMPA-REG OUTCOME [76]	CANVAS [77]	DECLARE-TIMI 58 [78]	VERTIS-CV [89]
Intervention	Empagliflozin	Canagliflozin	Dapagliflozin	Ertugliflozin
Population (n)	7020 patients with T2DM and established CV disease	10,142 patients with T2DM and established CV disease or ≥2 CV risk factors	17,160 patients with T2DM established CV disease or risk factors for atherosclerotic CV disease	8246 patients with T2DM and established CV disease
Established CV dis ease (%)	99	66	41	99
Primary outcome(s) (HR (95% CI) *p* value)	3P-MACE 0.86 (0.74–0.99) *p* = 0.04	3P-MACE 0.86 (0.75–0.97) *p* = 0.02	3P-MACE 0.93 (0.84–1.03) *p* = 0.17 CV death or hospitalization for HF 0.83 (0.73–0.95) *p* = 0.005	3P-MACE 0.97 (0.85–1.11) *p* < 0.001 for noninferiority
Key secondary out comes (HR (95% CI))	4P-MACE 0.89 (0.78–1.01)	All-cause mortality 0.87 (0.74–1.01) CV death 0.87 (0.72–1.06) Progression of albuminuria 0.73 (0.67–0.79) CV death or hospitalization for HF 0.78 (0.67–0.91)	≥40% decrease in eGFR to <60 mL/min/1.73 m^2^ or new end-stage renal disease or death from renal/CV cause 0.76 (0.67–0.87) All-cause mortality 0.93 (0.82–1.04)	CV death or hospitalization for HF 0.88 (0.75–1.03) CV death 0.92 (0.77–1.11) Renal death or dialysis/transplant or doubling of serum creatinine from baseline 0.81 (0.63–1.04)
Other secondary outcomes (HR (95% CI))				
CV death	0.62 (0.49–0.77)	-	0.98 (0.82–1.17)	-
All-cause mortality	0.68 (0.57–0.82)	-	-	0.93 (0.80–1.08)
Fatal or non-fatal Myocardial infarction	0.87 (0.70–1.09)	0.89 (0.73–1.09)	0.89 (0.77–1.01)	1.04 (0.86–1.26)
Fatal or non-fatal stroke	1.18 (0.89–1.56)	0.87 (0.69–1.09)	1.01 (0.84–1.21)	1.06 (0.82–1.37)
Hospitalization for heart failure	0.65 (0.50–0.85)	0.67 (0.52–0.87)	0.73 (0.61–0.88)	0.70 (0.54–0.90)

**Table 2:** Large-scale clinical trials of SGLT2 inhibitors in heart failure.

	DAPA-HF [117]	EMPEROR-Reduced [118]	EMPEROR-Preserved [137]	DELIVER [138]
Intervention	Dapagliflozin	Empagliflozin	Empagliflozin	Dapagliflozin
Population (n)	4744 patients with NYHA class II-IV HFrEF	3730 patients with NYHA class II-IV HFrEF	5988 patients with NYHA class II-IVHFpEF (LVEF >40%)	6263 patients with NYHA class II-IV HFmrEF and HFpEF
T2DM%	42%	50%	49.1%	44.8%
Primary outcome(s) (HR (95% CI) *p* value)	Worsening HF or CV death 0.74 (0.65–0.85) *p* < 0.001	Hospitalization for HF or CV death 0.75 (0.65–0.86) *p* < 0.001	Hospitalization for HF or CV death 0.79 (0.69–0.90) *p* < 0.001	Worsening HF or CV death 0.82 (0.73–0.92) *p* < 0.001
CV death	0.82 (0.69–0.98)	0.92 (0.75–1.12)	0.91 (0.76–1.09)	0.88 (0.74–1.05)
hospitalization or urgent HF visit	0.70 (0.59–0.83)	-	-	0.79 (0.69–0.91)
hospitalization for HF	0.70 (0.59–0.83)	0.69 (0.59–0.81)	0.71 (0.60–0.83)	0.77 (0.67–0.89)
urgent HF visit	0.43 (0.20–0.90)	-	-	0.76 (0.55–1.07)
Secondary outcomes (HR (95% CI))				
Cardiovascular death or heart-failure hospitalization	0.75 (0.65–0.85)	-	-	-
Total no. of hospitalizations for heart failure and cardiovascular deaths	0.75 (0.65–0.88)	-	-	-
Total no. of hospitalizations for heart failure	-	0.70 (0.58–0.85)	0.73 (0.61–0.88)	-
Total no. of worsening heart failure events and cardiovascular deaths	-	-	-	0.77 (0.67–0.89)
Change in KCCQ total symptom score at month 8	1.18 (1.11–1.26)	-	-	1.11 (1.03–1.21)
Death from any cause	0.83 (0.71–0.97)	0.92 (0.77–1.10)	1.00 (0.87–1.15)	0.94 (0.83–1.07)
Other outcomes (HR (95% CI)				
Change in quality-of-life score on KCCQ at week 52	-	1.7 (0.5–3.0)	1.32 (0.45–2.19)	-

T2DM: type 2 diabetes mellitus; CV: cardiovascular; HF: heart failure; LVEF: left ventricle ejection fraction; HR: hazard ratio; CI: confidence interval; KCCQ: Kansas City Cardiomyopathy Questionnaire; eGFR: estimated glomerular filtration rate.

## Data Availability

Not applicable.
